# Psychiatric Profile of Retinal Detachment Surgery under Regional Block

**DOI:** 10.4103/0974-9233.53368

**Published:** 2008

**Authors:** Emad Abboud, Afaf Mansour, Waleed Riad

**Affiliations:** 1 From the Department of Ophthalmology, King Khaled Eye Specialist Hospital, Riyadh, Saudi Arabia; 2 From the Department of Psychiatry, College of Medicine, Alexandria University, Egypt; 3 From the Department of Anesthesia, King Khaled Eye Specialist Hospital, Riyadh, Saudi Arabia

**Keywords:** anxiety, depression, retinal detachment, peribulbar anesthesia

## Abstract

**Purpose:**

The aim of this study was to investigate whether Saudi patients undergoing retinal surgery are more prone to perioperative anxiety and/or depression, to determine the relation between pre and postoperative emotional upset and also, to find the relation between severity of visual impairment and psychological dysfunction.

**Methods:**

Forty patients with retinal detachment (RD) undergoing Pars Plana Vitrecctomy were enrolled in this descriptive study. Regional block was performed using peribulbar technique in order to avoid confounding psychological effects of general anesthesia. The patients were tested for anxiety and depression using Hamilton Anxiety Rating Scale (HARS) and Beck Depression Inventory (BDI) one day before surgery and before discharge.

**Results:**

Psychological disturbance reported only by 17.5 percent of the studied patients. Preoperatively 71 percent of them showed mild to moderate anxiety. After the procedure, 80 percent of anxious patients maintained or experienced decrease level of anxiety. In addition to anxiety, 20 percent of anxious patient developed postoperative mild depression. 14 percent of the psychologically disturbed patients had moderate depression before surgery which became milder after it. Another 14 percent showed severe anxiety and moderate depression only postoperatively. Severe visual impairment was reported by 86 percent of psychological disturbed patients.

**Conclusion:**

Saudi patients with RD undergoing retinal procedures infrequently suffered anxiety and/or depression. Preoperative psychological disturbances were a good predictor of postoperative emotional upset. Perioperative psychological disturbances were related positively to the severity of visual impairment.

Seriously ill patients are frequently suffering from psy-chological disturbances secondary to their physical illness. These disturbances are usually in the form of depression and anxiety symptoms, which possibly acted as a defense against the threat of the disease.^1^ Vision provides the fundamental basis for social adjustment and normal psychological development. It has long been recognized that emotional disturbance accompanies visual loss.^2^ There is little information available concerning emotional distress among Saudi visually impaired individuals.

Patients undergoing surgery commonly experience anxiety. It is assumed that major surgery or that with unknown outcome produces more anxiety.^3^ Perioperative anxiety is also influenced by the patient's concern about his or her general health, uncertainty regarding the future, type of anaesthesia to be performed, post operative pain,^4^ loss of independence, and fear of death.^5^ Many patients also experience depressive symptoms presurgically which has been thought to increase after the operation.^6^ Researchers correlate between the degree of preoperative psychological stress and recovery, stressing the importance of emotional factors in treatment.^7^

The quality of life rather than longevity is a significant consideration for a human being. Ocular diseases have a major impact on quality of life because visual impairment potentially affects so many different aspects of functions.^8^

The primary aim of this descriptive study was to investigate whether patients with retinal detachment undergoing retinal surgery are more prone to perioperative anxiety and/or depression. The secondary aim was to determine the relation between pre and postoperative emotional upset and also, to find the relation between severity of visual impairment and psychological dysfunction.

## Methods

After obtaining the approval of hospital's research and human ethics committees and informed patient consent, forty Saudi adult patients of both sexes were enrolled in this descriptive study. All patients had retinal detachment and were scheduled for Pars Plana Vitrectomy under regional anesthesia in King khaled Eye Specialist Hospital. Exclusion criteria included patients with current use of any psychiatric medication or cognitive impairment that might affect the psychometric assessment. Subjects who were known to have a chronic uncontrolled disease such as cardiac disease, diabetes mellitus, cancer, cerebrovascular accident, and renal disease were also excluded. These diseases were selected because they have a major effect on the emotional status.^9^

Following a complete ophthalmic examination, primary ocular diagnosis, ocular involvement, duration of ocular disease, previous procedure and best-corrected visual acuity were recorded. Visual impairment was classified according to visual acuity (VA) into mild (VA > 20/44), moderate (VA from 20/44 to 20/125) and severe (VA < 20/125).^10^

Psychological assessment was performed by Beck Depression Inventory (BDI)^11^ revised version and Hamilton Anxiety Rating Scale (HARS)^12^ for depression and anxiety respectively. Those psychological tools had been chosen because they are the best-known survey instruments for identifying symptoms of depression and anxiety. They are easy to perform, time saving, results are easily scored, analyzed and recorded. These scales have been used in many diverse clinical settings as well as in general population surveys, and their validity and reliability have been demonstrated previously. They are adequate indicators for surgical related stress.^13^ The scales were conducted in-person by a research assistant who was trained well in the administration of these questionnaires. The patients were tested one day after admission and one day before discharge. For the scales to be conducted in the postoperative period, the patients had to be fully conscious and orientated to time and place. All cases were evaluated by a psychiatrist on the basis of the Structured Clinical Interview for DSM-IV (SCID-I).^14^

It is well known that general anesthesia causes postoperative cognitive dysfunction.^15^ In order to eliminate this effect, surgery was done under local anesthesia. Regional block was performed using Peribulbar technique. The anesthetic agent used in this study was a mixture of xylocaine 2% and bupivacine 0.5% 2:3 with 5 unit hayalourinase/ ml of anesthetic solution. After negative aspiration up to 10 ml of local anesthetic solution was injected.

## Results

The study was carried out on 40 Saudi patients. Socio-demographic variables and ophthalmic data are listed in Tables 1 and 2 respectively.

**Table 1:** Socio-demographic Data

Age (years)	61.7(10.6)

Sex	
Male	29 (72.5%)
Female	11 (27.5%)

Residence	
Urban	36 (90%)
Rural	4(10%)

Educational Level	
Illiterate	18(45%)
Grade 11 or less	9 (22.5%)
High school	4(10%)
Collage or higher degree	9 (22.5%)

Socioeconomic Status	
Very low– low	6(15%)
Low– middle	22 (55%)
Middle - High	12(30%)

Data expressed as a mean value (SD) or number and percentages.

**Table 2:** Ophthalmic Data

Diagnosis:	
Rhgmatogenous Retinal detachment	9 (22.5%)
Tractional Retinal detachment	31 (77.5%)

Ocular involvement:	
One eye	17(42.5%)
Two eyes	23 (57.5%)

Visual acuity:	
Mild	1 (2.5%)
Moderate	6 (15%)
Severe	33 (82.5%)

Duration of symptoms (years)	2.53 (0.8)

Previous procedure:	
Laser	25 (62.5%)
Surgery	10(25%)
None	5 (12.5%)

Data expressed as a mean value (SD) or number and percentages.

**Figure 1:**
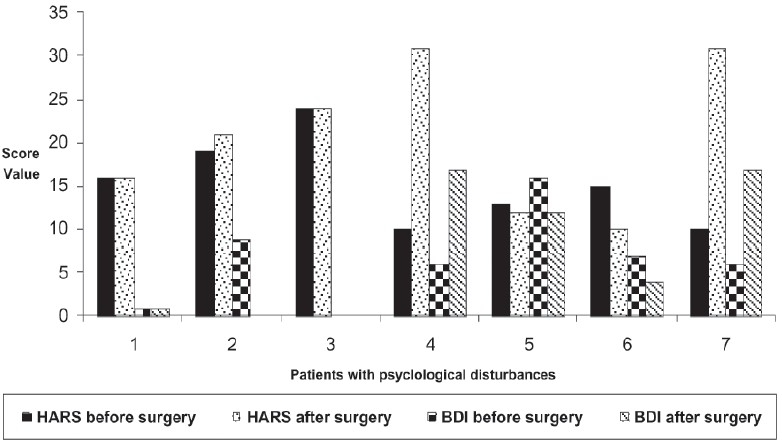
Psychological assessment. HARS: Hamilton Anxiety Rating Scale; BDI: Beck Depression Inventory scale

Patients with Psychological disturbances were displayed in Figure (1). They were only by 7 patients (17.5%) of the studied sample. Preoperatively 71% (5 patients) of them had mild to moderate anxiety. After the procedure, 80% (4 patients) of anxious patients maintained or experienced decreased level of anxiety. In addition to anxiety, 20% (1 patient) of anxious patients developed postoperative mild depression. From the psychologically disturbed patients 14% (1 patient) had moderate depression before surgery which became milder after surgery. Another 14% (1 patient) showed severe anxiety and moderate depression only postoperatively.

Moderate to severe visual impairment was reported by 97.5% (39 patients) of the studied population. However, severe visual impairment was documented in 86% (6 patients) of psychologically disturbed subjects.

## Discussion

This study showed lower incidence of perioperative anxiety and depression in Saudi patients with retinal detachment undergoing surgical procedures. Many patients with preoperative psychological disturbance retained variable degree of the disease postoperatively. Severe visual impairment is a common feature of most of psychologically disturbed patients.

Blumenfield and Thompson proposed that psychological responses exhibited by a patient- as a reaction to physical illness-depend on the nature and severity of the physical illness itself, the characteristic personality style and coping pattern. Also doctor and nurses responses to the patient modify his psychological reaction to illness and hospitalization.^16^

The present work showed that anxiety for the whole studied sample was 15% (6 patients) which is considered low compared to what was previously reported by other studies. Marantetes and Masur mentioned that the incidence of preoperative anxiety has been reported to reach 80% among adult patients.^17^ Moreover, Scott and his group demonstrated that emotional distress is more prevalent among patients with retinal disease than severely medically ill hospitalized and outpatients scheduled for audiological evaluation.^10^ They concluded that vision loss represents an additional significant risk factor for the emotional distress. Caumo and his group reported that preoperative anxiety correlate with high postoperative anxiety, increase postoperative pain and analgesic requirements.^18^ Lampic et al, reported that patients who do not express high levels of anxiety may be either truly less anxious or anxious but not giving overt expression to their emotions.^19^ Shafer et al, reported that male patients were reluctant to show their anxiety.^20^ The regional block probably produced superior postoperative analgesia both in terms of quality and uniformity compared to general anesthesia.^21^ All of the above could explain the reduced incidence of anxiety reported by our patients.

Severe level of depression but not anxiety was reported by Augustin et al, who proved that it was strongly associated with visual impairment.^22^ Also, Barcia and Psiquiatia highlighted the fact that in the blind the typical reaction is depression.^23^ This maladjustment could be due to difficulties in social functioning, changes in social support and loneliness.^23^ We expected to find more patients with depression. This low figure could be attributed to the nature of the culture to which the patients of this study belong. There are multiple factors, which might help the patient to cope with, and accept such serious diseases with their sequalae. First, the religious belief implicates the tendency to perceive and accept any stressful situations as a test of faith in and submission to God. On the other hand, facing such situations with feeling of anger and non-acceptance is sinful. Also, spiritual well-being gives patient more support and courage when facing stresses and difficulties.^24^ Second, the social support derived from the adherent social network in which these patients live have been frequently found to have positive effect.^25^ Third, denial as known defensive mechanism in face of dangerous situations like serious diseases, influence the quality of life by improving the sense of well-being.^26^ Lastly, Depression may not be also diagnosed because patients are often reluctant to report depressive symptoms to the treating team because they do not want to bother the nurses or physicians, or they fear being stigmatized by having mental illness especially in our Arabic societies.^27^,^28^

In this study, about 43% of those patients who had preoperative psychological disturbances showed a reasonable degree of improvement postoperatively. This was consistent with the findings of Schumacher et al and Giovagnoli et al, who reported the same observation for patient with leukemia and brain tumor.^29^,^30^ They have explained this improvement by the increased acceptance and adaptability of the patients to the disease. On the other hand, contradicting results was shown by Anderson;^31^ however, his patients had serious illness of rapid progressive nature.

This study has been done on a heterogeneous group of patients in regards of their socio-demographic background. Many cultural, social and educational factors need to be evaluated in a more extensive view. However, the clinical impressions gave rise to many questions, which stimulate further studies of larger samples in this field. It is worth to note that evaluating the mental status of ophthalmic patients while planning their management will help to provide the optimal treatment.

## Conclusion

Saudi patients with RD undergoing retinal procedures infrequently suffered from perioperative anxiety and/or depression. This could be attributed to the religious belief, cultural bases, social support and denial as a defensive mechanism. Preoperative psychological disturbances were a good predictor of postoperative emotional upset. Severe visual impairment related positively to the perioperative psychological disturbances.
